# Effect of Loss of Teeth and its Association with General Quality of Life using Geriatric Oral Health Assessment Index (Gohai) among Older Individuals Residing in Rural Areas

**Published:** 2017-03

**Authors:** Vinaya Kundapur, Rakshith Hegde, Manoj Shetty, Sunil Mankar, Mohammed Hilal, Hari Prasad A

**Affiliations:** 1 Department of Prosthodontics,M R Ambedker Dental College & Hospital, Bangalore, India;; 2 Department of Prosthodontics, A. B. Shetty Memorial Institute of Dental Sciences, Deralakatte, Mangalore, India;; 3 Department of conservative & Endodontics, DA Pandu Memorial RV Dental College, Bangalore, India

**Keywords:** Geriatric, Dentures, Edentulous

## Abstract

The development of measures for assessing oral health status is essential to the evolution and maturation of a scientific knowledge based in geriatric dentistry. Their development as branches of clinical care depends in part as ability to demonstrate an effective grasp of the problems of elderly and their solutions. Any strategy for altering the health status of elderly requires a technology for first assessing that health status and then detecting increments of progress. Development of geriatric oral health assessment index (GOHAI) is a self reported measure designed to assess the oral health problems of older individuals. The goal of geriatric assessment is to collect information that will facilitate diagnosis and suggest appropriate interventions. Such a measure would facilitate estimation of prevalence of oral functional problems in older individuals, It would also increase understanding of psychological impact of oral disease and provide a means for comparing the effectiveness of various dental treatment. It also emphasizes on social well being and reflects quality of life following replacement of missing teeth.

## BRIEF RESUME OF THE INTENDED WORK

### Need for the survey

The development of measures for assessing oral health status is essential to the evolution and maturation of a scientific knowledge based in geriatric dentistry. Their development as branches of clinical care depends in part as ability to demonstrate an effective grasp of the problems of elderly and their solutions. Any strategy for altering the health status of elderly requires a technology for first assessing that health status and then detecting increments of progress.

Development of geriatric oral health assessment index (GOHAI) (1) a self reported measure designed to asses the oral health problems of older individuals. The goal of geriatric assessment is to collect information that will facilitate diagnosis and suggest appropriate interventions. Such a measure would facilitate estimation of prevalence of oral functional problems in older individuals, it would also increase understanding of psychological impact of oral disease and provide a means for comparing the effectiveness of various dental treatment modalities in older adults.

The geriatric oral health assessment index (GOHAI), guidelines suggest use of measure to asses the impact of oral condition on quality of life (QoL) of individual. The 12 item GOHAI was developed to evaluate three dimensions of oral health related quality of study (QoL).

The purpose of this study was to test a self reported oral health assessment index for use in older adult population in kundapura taluq. GOHAI was guided by several underlying assumptions:

1. Oral health can be measured using patients self reports;
2. Levels of oral health vary among patients, this variation can be measured using patients self reported measure;
3. Predictors of oral health self reports can be identified.

## REVIEW OF THE LITERATURE

In a study to translate and validate the Geriatric Oral Health Assessment Index (GOHAI into the Malay language for use in Malaysia. GOHAI demonstrated acceptable validity and reliability and will be an important instrument to measure oral health-related quality of life among Malay-speaking Malaysians. Use of the Malay language version GOHAI should also be pursued among diverse adult age groups.

A study was done to asses oral health related quality of life among patients with complete denture one month before and one month after insertion using GOHAI. The study concluded that patients showed improvement in functional changes after placement of complete dentures (3).

A study was conducted to evaluate the impact of the placement of complete dentures by using the GOHAI, Oral health quality of life indicators can be used to evaluate the effects of dental treatments. The study concluded that The GOHAI can be used to evaluate needs for and effect of the making of new complete dentures.

A study was done to translate the original English version of the Geriatric Oral Health Assessment Index (GOHAI) into Arabic and assess its validity and reliability for use among people in North Jordan. The study concluded that GOHAI demonstrated acceptable validity and reliability when used for people in North Jordan. It could therefore be used as a valuable instrument for measuring oral health-related quality of life for people.

A study was done by Translation, reliability analysis and validation of a German version of the Geriatric/General Oral Health Assessment Index (GOHAI) The responsiveness to change in oral health status was assessed by pre- and post-treatment comparison The German version of the GOHAI had sufficient reliability, validity and responsiveness to be used as measure of oral health-related quality of life in cross-sectional and longitudinal studies of the elderly.

### Objectives of the study

To evaluate three dimensions of oral health related quality of life (QoL).

1. Physical function including speech, eating and swallowing;
2. Psychological function including worry / concern about oral health, dissatisfaction with appearance, self conscious about oral health and avoidance of social contact because of oral problems;
3. Pain and discomfort including use of medication to relieve pain or discomfort from mouth (2).

## MATERIALS AND METHODS

### Source of the data

The study was conducted to evaluate oral health related quality of life in relation to physical functions, psychological function, pain and discomfort in older individuals residing in rural areas of kundapura taluq, udupi district, karnataka, India.

All subjects were between 60 to 90 years.

### Method of collection of data

The present study was a questionnaire survey. The questionnaire had 16 questions, 4 on physical functions, 5 on psychological, 3 on pain and discomfort. Patient consent was obtained before administration of questionnaire. Data included age, gender, education level, perceived need for dental treatment wears a denture, perceived general health and mean GOHAI score was obtained using the same questionnaire (Annexure 1).

The data collected were categorical and statistically analyzed using SPSS 15 version. The descriptive of age and mean GOHAI score was assessed. The frequencies of sex, education level, perceived need for dental treatment wears a denture, perceived general health was obtained. Chi square test was applied to see the association between mean GOHAI SCORE with age, sex, education level, perceived need for dental treatment wears a denture, and perceived general health was obtained.

## RESULTS

**Figure 1:**
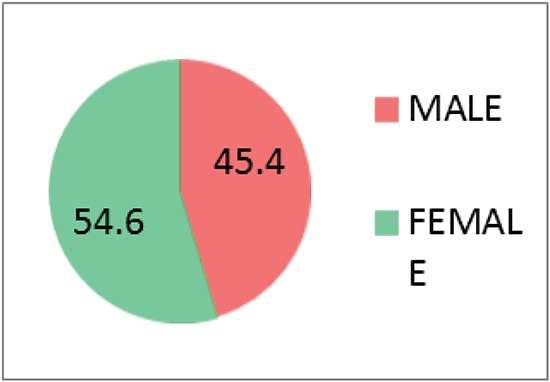


**Figure 2:**
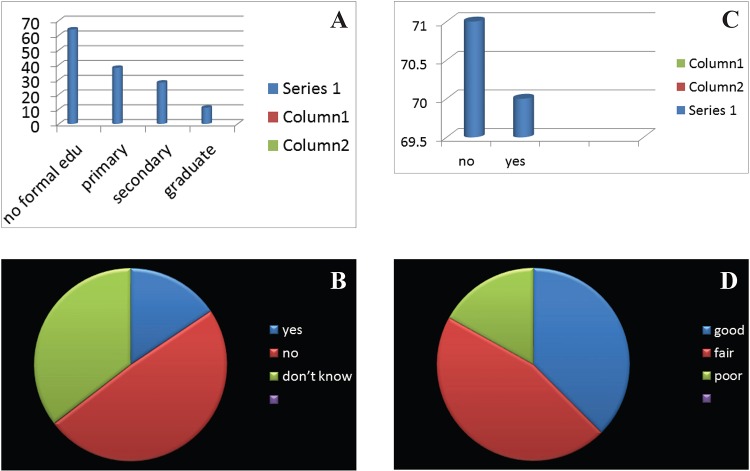
A) Frequency of education level; B) Frequency of percieved need for dental treatment; C) Wears prosthesis; D) Percieved general health.

**Figure 3:**
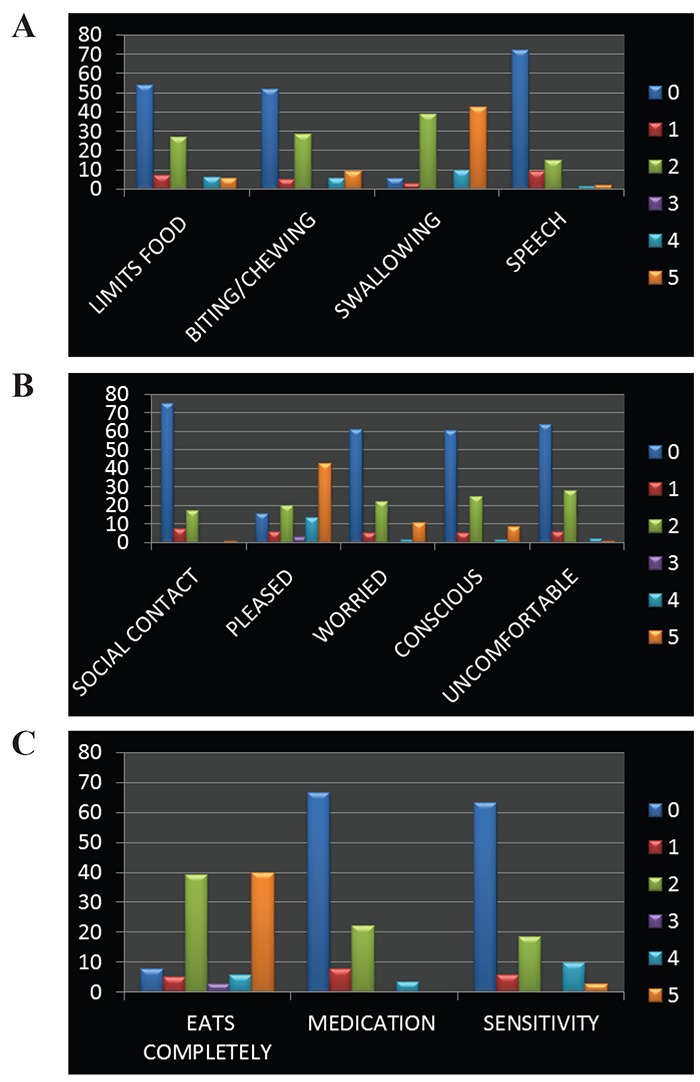
A) Physical Functions; B) Psycological; C) Pain and Discomfort.

A total of 141 subjects were included in this study, out of 141 respondents 64 were males and 77 were females (45.4% male, 54.6% females) (Figure 1). Mean age was 67.2 (SD 6.55, range 60-84 years). Subject characteristics are shown in Figure 2. Mean GOHAI sore of these study subjects was 17.58 and with the range of 2-40 and standard deviation of 7.322. The frequencies of, education level, perceived need for dental treatment, wears prosthesis, perceived general health was obtained (Table 1a, 1b, 1c, 1d) response to the GOHAI items tended to never, sometimes, or always with few utilizing in between responses (Table 2, Figure 3). 

The results of the chi square test between,

Mean GOHAI score and age

(p=0.000 and chi square value =836.406)

Mean GOHAI score and sex

(p=0.009 and chi square value =48.688)

Mean GOHAI score and education

(p=0.000 and chi square value =138.332)

Mean GOHAI score and treatment need

(p=0.001 and chi square value =95.925)

Mean GOHAI score and wears prosthesis

(p=0.005 and chi square value =51.368)

Mean GOHAI score and general health

(p=0.012 and chi square value =116.006)

**Table 1a:** Frequencies of education level

	Frequency	Percent

No formal education	64	45.4
Primary	38	27.0
Secondary	28	19.9
Graduate	11	7.8
Total	141	100.0

**Table 1b:** Frequencies of perceived need for dental treatment of subjects

	Frequency	percent

No	22	15.6
Yes	69	48.9
Don’t know	50	35.5
Total	141	100.0

**Table 1c:** Frequencies of wears prosthesis

	Frequency	percent

No	71	50.4
Yes	70	49.6
Total	141	100.0

**Table 1d:** Frequencies of perceived general health

	Frequency	Percent

good	56	39.7
Fair	62	43.4
poor	23	63.3
Total	141	100.0

**Table 2:** Percentage distribution of subjects on individual GOHAI items

**Table 2A. Limits food**		

	**Frequency**	**percent**

0	76	53.9
1	10	7.1
2	38	27.0
4	9	6.4
5	8	5.7

**Table 2b. Biting/chewing**		

	**Frequency**	**percent**

0	73	51.8
1	7	5.0
2	40	28.4
4	8	5.7
5	13	9.2

**Table 2c. Swallowing**		

	**Frequency **	**percent**

0	8	5.7
1	4	2.8
2	55	39.0
4	14	9.9
5	60	42.6

**Table 2d. Speech**		

	**Frequency**	**percent**

0	102	72.3
1	13	9.2
2	21	14.9
4	2	1.4
5	3	2.1

**Table 2e. Social contact**		

	**Frequency**	**percent**

0	106	75.2
1	10	7.1
2	24	17.0
5	1	0.7

**Table 2f. Pleased**		

	**Frequency**	**percent**

0	22	15.6
1	8	5.7
2	28	19.9
3	4	2.8
4	19	13.5
5	60	42.6

**Table 2g. Worried**		

	**Frequency**	**percent**

0	86	61.0
1	7	5.0
2	31	22.0
4	2	1.4
5	15	10.6

**Table 2h. Conscious**		

	**Frequency**	**percent**

0	80	60.3
1	7	5.0
2	35	24.8
4	2	1.4
5	12	8.5

**Table 2i. Uncomfortable**		

	**Frequency**	**percent**

0	90	63.8
1	8	5.7
2	39	27.7
4	3	2.1
5	1	0.7

**Table 2j. Eats completely**		

	**Frequency**	**percent**

0	11	7.8
1	7	5.0
2	55	39.0
3	4	2.8
4	8	5.7
5	56	39.7

**Table 2k. Medication**		

	**Frequency**	**percent**

0	94	66.7
1	11	7.8
2	31	22.
4	5	3.5

**Table 2l. Sensitivity**		

	**Frequency**	**percent**

0	89	63.1
1	8	5.7
2	26	18.4
4	14	9.9
5	4	2.8

## DISCUSSION

In a study to translate and validate the Geriatric Oral Health Assessment Index (GOHAI into the Malay language for use in Malaysia. The 6-Likert scale GOHAI was translated into the Malay language and self-administered on 189 subjects aged 60+. All subjects underwent oral status assessment. The measure was assessed for construct and discriminant validity, for test-retest reliability and principal component factor. Mean GOHAI score was 46.2 (SD 9.7, range 17-60). The Cronbach’s alpha was 0.79. Mean GOHAI scores increased with more positive self-rated oral health and general health. The elderly with no perceived dental treatment need had higher mean GOHAI scores than those with perceived needs. There were slightly stronger inverse correlations between GOHAI scores and caries experience, number of teeth present, and number of pathologically mobile teeth. The measure demonstrated strong test-retest reliability. Using ANCOVA, self-rated perception of oral health and perceived need for dental treatment had the most significant impact on the GOHAI score.

In a study to translate the original English version of the Geriatric Oral Health Assessment Index (GOHAI) into Arabic and assess its validity and reliability for use among people in North Jordan. After translation into Arabic and back-translation to check the translation quality, a total of 288 participants completed the Arabic version of the GOHAI questionnaire. Individual GOHAI items were recoded and summed as originally recommended. The questionnaire sought information about socio-demographic characteristics and self-reported perception of general and oral health. Clinical examination included assessment of periodontal status, and number of decayed teeth, missing teeth, filled teeth and crowned teeth. Reliability, internal consistency, and concurrent, convergent and discriminant validity of GOHAI scores were examined. Mean GOHAI score was 40.9 (SD=10.6, range: 12 to 60). The test-retest correlation coefficient for add-GOHAI scores was 0.72, indicating good stability. Add-GOHAI scores increased with poorer perceived general and oral health. The Arabic translation of the GOHAI demonstrated acceptable validity and reliability when used for people in North Jordan. It could therefore be used as a valuable instrument for measuring oral health-related quality of life for people.

In a study in Assessment of changes in oral health-related quality of life among patients with complete denture before and 1 month post-insertion using Geriatric Oral Health Assessment Index As there is scanty literature available on GOHAI in the Indian population, the present study was undertaken to assess the changes in GOHAI before and 1 month after placement of dentures in completely edentulous patients reporting to a dental hospital at Indore, India. The GOHAI questionnaire was completed by the examiner who interviewed the patients (n=35) before placement of complete dentures and 1 month later. Mean, median values were calculated and the data were analysed using Wilcoxon signed-rank test. When overall mean was considered, the GOHAI scores increased from 27.48 to 30.19 (p=0.002; highly significant).Patients reported improvement in functional changes after placement of complete dentures.

## Annexure 1

**Table:**

**SL. NO: NAME: ADDRESS: AGE: SEX:**
**EDUCATIONAL LEVEL:**
A) NO FORMAL EDUCATION B) PRIMARY C) SECONDARY D) GRADUATE
**PRECIEVED NEED FOR DENTAL TREATMENT:**
A) NO B) YES C) DON'T KNOW
**WEARS A REMOVABLE COMPLETE /PARTIAL DENTURE:**
A) NO B) YES
**PRECIEVED GENERAL HEALTH:**
A) GOOD B) FAIR C) POOR
**PHYSICAL FUNCTIONS**
***1) Limit the kind of food***
A) NEVER (0) B) SELDOM (1) C) SOMETIMES (2) D) often (3) E) very often (4) F) always (5)
***2) Trouble in biting / chewing***
A) NEVER (0) B) Seldom (1) C) sometimes (2) D) often (3) E) very often (4) F) always (5)
***3)ABLE TO SWALLOW COMPLETELY***
A) NEVER (0) B) SELDOM (1) C) SOMETIMES (2) D) often (3) E)very often (4) F) always (5)
***4) Unable to speak clearly***
A) NEVER (0) B) SELDOM (1) C) SOMETIMES (2) D) often (3) E)very often (4) F) always (5)
**PSYCOLOGICAL**
***5) LIMIT CONTACTS WITH PEOPLE***
A) Never (0) B) seldom (1) C) sometimes (2) D) often (3) E)very often (4) F) always (5)
***6) Pleased with look of teeth***
A) Never (0) B) seldom (1) C) sometimes (2) D) often (3) E) very often (4) F) always (5)
***7) Worried about teeth , gums or dentures***
A) NEVER (0) B) SELDOM (1) C) SOMETIMES (2) D) often (3) E) very often (4) F) always (5)
***8) SELF CONSCIOUS OF TEETH, GUMS OR DENTURES***
A) NEVER (0) B) SELDOM (1) C) SOMETIMES (2) D) often (3) E) very often (4) F) always (5)
***9) UNCOMFORTABLE EATING IN FRONT OF OTHERS***
A) NEVER (0) B) Seldom (1) C) sometimes (2) D) often (3) E) very often (4) F) always (5)
**PAIN & DISCOMFORT**
***10) ABLE TO EAT COMPLETELY***
A) NEVER (0) B) SELDOM (1) C) SOMETIMES (2) D) often (3) E) very often (4) F) always (5)
***11) Used medication to relieve pain***
A) NEVER (0) B) SELDOM (1) C) SOMETIMES (2) D) often (3) E) very often (4) F) always (5)
***12) Sensitive to hot /cold / sweet food***
A) NEVER (0) B) Seldom (1) C) sometimes (2) D) often (3) E) very often (4) F) always (5)
